# Performance comparison of modified ComBat for harmonization of radiomic features for multicenter studies

**DOI:** 10.1038/s41598-020-66110-w

**Published:** 2020-06-24

**Authors:** R. Da-ano, I. Masson, F. Lucia, M. Doré, P. Robin, J. Alfieri, C. Rousseau, A. Mervoyer, C. Reinhold, J. Castelli, R. De Crevoisier, J. F. Rameé, O. Pradier, U. Schick, D. Visvikis, M. Hatt

**Affiliations:** 1 INSERM, UMR 1101, LaTIM, University of Brest, Brest, France; 2 Department of Radiation Oncology, Institut de cancérologie de l’Ouest René-Gauducheau, Saint-Herblain, France; 3 Radiation Oncology Department, University Hospital, Brest, France; 4 Department of Nuclear Medicine, University of Brest, Brest, France; 5 Department of Radiation Oncology, McGill University Health Centre, Montreal, Quebec Canada; 6 Department of Nuclear Medicine, Institut de cancerologie de l’Ouest René-Gauducheau, Saint-Herblain, France; 7 Department of Radiology, McGill University Health Centre, Montreal, Canada; 8 Radiotherapy Department Cancer, Institute Eugene Marquis, Rennes, France; 9 University of Rennes 1, LTSI, Rennes, France; 10 Department of Medical Oncology, Centre Hospitalier de Vendee, La Roche sur Yon, France; 11 CRCINA, University of Nantes, INSERM UMR1232, CNRS-ERL6001 Nantes, France

**Keywords:** Computational science, Scientific data, Statistics, Data integration, Image processing, Machine learning, Statistical methods

## Abstract

Multicenter studies are needed to demonstrate the clinical potential value of radiomics as a prognostic tool. However, variability in scanner models, acquisition protocols and reconstruction settings are unavoidable and radiomic features are notoriously sensitive to these factors, which hinders pooling them in a statistical analysis. A statistical harmonization method called ComBat was developed to deal with the “batch effect” in gene expression microarray data and was used in radiomics studies to deal with the “center-effect”. Our goal was to evaluate modifications in ComBat allowing for more flexibility in choosing a reference and improving robustness of the estimation. Two modified ComBat versions were evaluated: M-ComBat allows to transform all features distributions to a chosen reference, instead of the overall mean, providing more flexibility. B-ComBat adds bootstrap and Monte Carlo for improved robustness in the estimation. BM-ComBat combines both modifications. The four versions were compared regarding their ability to harmonize features in a multicenter context in two different clinical datasets. The first contains 119 locally advanced cervical cancer patients from 3 centers, with magnetic resonance imaging and positron emission tomography imaging. In that case ComBat was applied with 3 labels corresponding to each center. The second one contains 98 locally advanced laryngeal cancer patients from 5 centers with contrast-enhanced computed tomography. In that specific case, because imaging settings were highly heterogeneous even within each of the five centers, unsupervised clustering was used to determine two labels for applying ComBat. The impact of each harmonization was evaluated through three different machine learning pipelines for the modelling step in predicting the clinical outcomes, across two performance metrics (balanced accuracy and Matthews correlation coefficient). Before harmonization, almost all radiomic features had significantly different distributions between labels. These differences were successfully removed with all ComBat versions. The predictive ability of the radiomic models was always improved with harmonization and the improved ComBat provided the best results. This was observed consistently in both datasets, through all machine learning pipelines and performance metrics. The proposed modifications allow for more flexibility and robustness in the estimation. They also slightly but consistently improve the predictive power of resulting radiomic models.

## Introduction

Recent advancements in radiomics, the process consisting of the extraction of vast arrays of quantitative features using high-throughput computing from medical images such as magnetic resonance (MR), computed tomography (CT), and positron emission tomography (PET)^1–4^, leads to large quantitative sets of features made available to both research and clinical communities for investigation of potential impact in the clinical setting^5^. For oncological applications in particular a number of radiomics-driven prognostic/predictive studies related to numerous cancer types have shown promising results with potential applications for personalized medicine^6^.

In order to generate convincing results regarding the potential clinical value of radiomics as a prognostic tool, it is essential to consider large patient cohorts that can be only available through multicenter studies^7–10^. Indeed, most radiomics studies to date have been single-center based and retrospective in nature, in small cohorts of patients, and most radiomic models are not externally validated^11,12^. One of the main benefits of multicenter studies lies in the higher statistical relevance of the results obtained in larger datasets of different samples which naturally leads to more robust inference. However, multicenter radiomic studies are challenging, as gathering data from several centers for a centralized analysis is complex for legal, ethical, administrative and technical reasons. Irrespective of whether or not data and images are stored/analyzed in a centralized manner, variability in scanner models, acquisition protocols and reconstruction settings are unavoidable in the current clinical practice. Yet radiomic features are notoriously sensitive to such variations, which subsequently hinders pooling data to carry out statistical analysis and/or machine learning (ML) in order to build robust models^13–16^. Hence, there is a clear need for the harmonization of features in order to allow consistent findings in radiomics multicenter studies^17^. Within this context, there are two main approaches to address this issue: (i). harmonizing images and (ii). harmonizing radiomic features.

The first approach addresses the harmonization issue in the image domain and usually considers standardization of acquisition protocols and reconstruction settings, relying for example on guidelines already available for PET/CT imaging^18,19^. It has been shown recently that although such an approach can help towards reducing multicenter effects, it may still be insufficient to fully compensate them^19^. More recently, techniques based on generative adversarial networks have also been considered in order to generate images with more similar properties^20,21^. The second approach addresses the issue in the feature domain by either selecting features prior to the statistical analysis based on their robustness in order to rely only on features insensitive to multicenter variability, or by keeping all features and harmonizing their statistical properties so they can be pooled during the modeling step. Within this context different methods have been considered, such as normalization^22^ or batch-effect correction using the ComBat method^23^.

In this study, our objective was to develop and evaluate hybrid techniques based on ComBat to allow for more flexibility in the choice of a reference, and improved robustness in the estimation of the required transform.

## Materials and Methods

### ComBat approach description

ComBat consists in dealing with the variability of parameters’ distributions so they can be pooled together. The method was initially described in genomics^24^, where the so-called “batch effect” is the source of variations in measurements caused by handling of samples by different laboratories, tools and technicians. This “batch effect” is conceptually similar to variations induced in radiomic features by the scanner model, the acquisition protocol and/or the reconstruction settings, sometimes called “center effect”. ComBat identifies a batch-specific transformation to express all data in a common space devoid of center effects. A modified version called M-ComBat which centers the data to the location and scale of a pre-determined “reference” batch was later introduced^25^. ComBat eliminates batch effects primarily based on an empirical Bayes framework. It has demonstrated robustness with smaller sample sizes^26^, and continues to be a widely used approach^27–29^. ComBat was observed as being “*best able to reduce and remove batch effects while increasing precision and accuracy*” when compared to five other popular batch effect removal methods^26^. ComBat within the context of radiomic features harmonization works by first standardizing them according to:

1 $${Z}_{ijg}=\frac{{Y}_{ijg}-{\widehat{\alpha }}_{g}-X{\widehat{\beta }}_{g}}{{\widehat{\sigma }}_{g}}$$

where ordinary least-squares is used to calculate features-wise mean and standard deviation estimates, ${\widehat{\alpha }}_{g}$ and ${\widehat{\sigma }}_{g}$, across feature *g*, sample *j*, and center *i*. *Y*_*ijg*_ refers to the raw radiomics features values, and $X{\widehat{\beta }}_{g}$ represents potential non-center related covariates and coefficients in the model. The standardized data is assumed to be normally distributed ${Z}_{ijg} \sim N({\gamma }_{ig},{\delta }_{ig}^{2})$, where *γ*_*ig*_ and ${\delta }_{ig}^{2}$ are the center effect parameters with Normal and Inverse Gamma prior distributions, respectively. The method of moments is used to estimate hyperparameters which are used to compute the empirical Bayes estimates of conditional posterior means features-wise by center for the center effects parameters. The final center effect adjusted values are given by

2 $${Y}_{ijg}^{\ast }=\frac{{\widehat{\sigma }}_{g}}{{\widehat{\delta }}_{ig}^{\ast }}({Z}_{ijg}-{\widehat{\gamma }}_{ig}^{\ast })+{\widehat{\alpha }}_{g}+X{\widehat{\beta }}_{g}$$

One of the limitations of ComBat is that it centers the data to the overall, grand mean of all samples, which results in an adjusted data matrix that is shifted to an arbitrary location that no longer coincides with the location of any of the original centers. This can lead to harmonized features losing their original physical meaning (and impossible values, *e.g*., negative volumes or SUV).

### M-ComBat

A modified version of ComBat (M-ComBat) previously proposed^25^ shifts samples to the mean and variance of the chosen reference batch, rather than the grand mean and pooled variance. This is achieved by changing the standardizing mean and variance of the estimates, ${\widehat{\alpha }}_{g}$ and ${\widehat{\sigma }}_{g}$, to center-wise estimates, ${\widehat{\alpha }}_{ig}$ and ${\widehat{\sigma }}_{ig}$, such that the standardized values are then given by

3 $${Z}_{ijg}=\frac{{Y}_{ijg}-{\widehat{\alpha }}_{ig}-X{\widehat{\beta }}_{g}}{{\widehat{\sigma }}_{ig}}$$

Furthermore, the mean and variance estimates used in the final center effect adjusted data are calculated using the feature-wise mean and variance estimates of the ‘gold-standard’, reference center, *i* = *r*.

The M-ComBat adjusted data are then given by

4 $${Y}_{ijg}^{\ast }=\frac{{\widehat{\sigma }}_{i=r,g}}{{\widehat{\delta }}_{ig}^{\ast }}({Z}_{ijg}-{\widehat{\gamma }}_{ig}^{\ast })+{\widehat{\alpha }}_{i=r,g}+X{\widehat{\beta }}_{g}$$

### Proposed modifications: B-ComBat and BM-ComBat

We propose a parametric bootstrap addition for the parameters in the ComBat and M-ComBat models, respectively. Here, we will use the bootstrap to construct a robust estimate and test improvement of the predictive ability of the models. Thus, from the estimates obtained in Eqs. (2) and (4), we perform the following:

1. From the initial estimates obtained in the fitted ComBat and M-ComBat, resample ***B*** = **1000** times with replacement.
2. Fit each resamples in the ComBat and M-ComBat models to obtain the *B* estimates of the coefficients, denoted by ${\gamma }_{ig}^{\ast }$, ${\alpha }_{g}^{\ast }$, ${\alpha }_{i=r,g}^{\ast }$, ${\beta }_{g}^{\ast }$ and ${\delta }_{ig}^{\ast }$.
3. Compute the final estimates of the coefficients using Monte Carlo method by getting the mean of the ***B*** estimates, ${\gamma }_{igk}^{\ast }={\sum }_{i}^{B}\,\frac{{\gamma }_{ig}^{\ast }}{k}$, ${\alpha }_{igk}^{\ast }={\sum }_{i}^{B}\,\frac{{\alpha }_{ig}^{\ast }}{k}$, ${\alpha }_{i=r,g}^{\ast }={\sum }_{i}^{B}\,\frac{{\alpha }_{i=r,g}^{\ast }}{k}$, ${\beta }_{g}^{\ast }={\sum }_{i}^{B}\,\frac{{\beta }_{ig}^{\ast }}{k}$, and ${\delta }_{ig}^{\ast }={\sum }_{g}^{B}\,\frac{{\delta }_{ig}^{\ast }}{k}$ where ***k*** = **1, …**, ***B***.

Hence, the final *B-ComBat* and *BM-ComBat* bootstrapped adjusted data are given respectively by

5 $${Y}_{ijg}^{B-ComBat}=\frac{{y}_{ijg}-{\widehat{\alpha }}_{gk}-{X}_{ij}{\widehat{\beta }}_{gk}-{\gamma }_{igk}^{\ast }}{{\delta }_{igk}^{\ast }}+{\widehat{\alpha }}_{gk}+{X}_{ij}{\widehat{\beta }}_{gk}$$

6 $${Y}_{ijg}^{BM-ComBat}=\frac{{y}_{ijg}-{\widehat{\alpha }}_{(i=r,g)k}-{X}_{ij}{\widehat{\beta }}_{gk}-{\gamma }_{igk}^{\ast }}{{\delta }_{igk}^{\ast }}+{\widehat{\alpha }}_{(i=r,g)k}+{X}_{ij}{\widehat{\beta }}_{gk}$$

### Datasets: patient cohorts, imaging and clinical endpoints

#### Locally advanced cervical cancer (LACC)

A cohort of 197 patients with locally advanced cervical cancer from 3 centers (Brest, n = 119 and Nantes, n = 50, in France, and Montreal, n = 28, in Canada) was exploited. Fluorodeoxyglucose (FDG)-PET, post-injection gadolinium contrast-enhanced MRI (GADO), T2-weighted MRI (T2) and apparent diffusion coefficients (ADC) maps from diffusion-weighted MRI were available for the radiomics pipeline. PET/CT and MRI settings were the same for all patients in each specific center (see Supplemental Table 1). Treatment consisted of curative radiotherapy (RT, external and brachytherapy) and chemotherapy. Data were retrospectively collected and a minimum follow-up of one year was ensured. Prediction of local failure (LF) was chosen as the endpoint, as an assessment at the time of diagnosis of the likelihood of LF would provide a rationale to adapt treatment (*e.g*., avoid systemic treatment for patients with low risk of recurrence)^30^. In this case, labels to rely upon for ComBat harmonization were well-defined and known, as all patients within a given center had the same acquisition and reconstruction settings applied with the same scanner.

#### Locally advanced laryngeal cancer (LALC)

A cohort of 98 patients with histologically proven locally advanced laryngeal or hypopharyngeal cancer who were treated with laryngeal preservation using induction chemotherapy regimen (a combination of docetaxel, cisplatin and 5-fluorouracil, TPF) from 5 French centers (Brest, Nantes, Rennes, La Roche-sur-Yon and Quimper) was exploited. All patients had a contrast-enhanced computed tomography (CE-CT) at diagnosis. Response to chemotherapy was assessed after 2 or 3 cycles depending on centers and was based on clinical examination (endoscopic evaluation of larynx mobility) and imaging evaluation (CT scan and/or FDG PET/CT). The chosen primary endpoint is the lack of response to TPF, defined as a non-remobilization of the larynx if laryngeal mobility was decreased or abolished at diagnosis, or a response of the primary tumour <50% (RECIST criteria). This endpoint was chosen, as it significantly modifies the subsequent therapy for patients. Non-responders to TPF were indeed referred for a (pharyngo-)laryngectomy followed by postoperative RT, whereas responders could keep their larynx and received conservative RT with or without chemotherapy, depending on pathological risk factors. In contrast to the LACC case, a great variability was observed in terms of scanner models, collimation/acquisition settings and reconstruction parameters, even within each of the five centres (see Supplemental Table 2). It was thus challenging to set labels manually and it would lead to apply ComBat with an unrealistic number of labels (at least 15 for 98 patients). Hence, we implemented a solution that allows for automatic identification of cluster of patients with similar radiomic features, as well as the optimal number of clusters (see section “Unsupervised hierarchical clustering with silhouette scoring”).

### Radiomics: feature extraction

In the present work, only radiomic features extracted from the images and the available clinical factors were exploited. All image analysis steps including tumor delineation and features extraction are summarized below. Primary tumour volume-of-interests (VOIs) were delineated by one specialist (F. Lucia in the LACC cases and I. Masson in the LALC cases) as corresponding to the gross tumour volume (GTV). VOIs were delineated on every scan independently. PET images were contoured automatically with the fuzzy locally adaptive Bayesian (FLAB) algorithm^31^ and all CT and MR images, manually with the 3D SlicerTM software^32^. For each VOI, 79 morphological and intensity-based features, as well as 94 textural features were extracted in 3D according to the most up-to-date reference document of the Image Biomarker Standardization Initiative (IBSI)^33^ and validated with respect to the consensus values^34^. Each of the 94 textural features was computed according to two grey-level discretization algorithms: “fixed bin number” (FBN) or “fixed bin width” (FBW). Regarding FBN, 64 bins were considered. For FBW, in the case of PET, width values of 0.25, 0.5, 1 and 2 standardized uptake values (SUVs) were considered, whereas in the case of CT, 10 or 25 Hounsfield Units (HUs) were considered. Texture matrices were built according to the merging strategy (by summation of 13 matrices calculated in each direction before texture calculation). The list of the extracted parameters are available in Supplemental Table 3.

### Experiments and analysis

All versions of ComBat described in sections A, B and C above were applied using the non-parametric version of ComBat, using the labels defined either as the 3 clinical centers for LACC or the labels obtained through unsupervised hierarchical clustering with silhouette scoring^35,36^ in the case of LALC (see “Unsupervised hierarchical clustering with silhouette scoring”).

The four different versions of ComBat were evaluated in terms of the resulting coefficient of variation (COV) in the harmonized features, compared to the untransformed variables. Radiomic features were then compared with ANOVA in terms of their statistical distributions across labels before and after harmonization with the four ComBat versions. In order to further evaluate the impact of harmonization, principal components analysis (PCA) was performed. Finally, the impact of the different ComBat versions on the performance of multiparametric models relying on radiomic features (and clinical factors) to predict the endpoints described previously was evaluated.

In LACC, the Brest data (n = 119, 40 LF) was used as the training set (and reference for M-ComBat), whereas the testing set was built by combining Nantes and McGill data (n = 78, 27 LF). In LALC, unsupervised hierarchical clustering was performed to automatically determine the optimal number of clusters to use in ComBat (see Supplemental data and details below). The cluster with the largest number of patients was chosen as the reference (as far as M-ComBat was concerned) and training set, whereas the other was chosen as the testing set.

Models predicting endpoints (as a binary task) for both datasets were built using 3 different ML pipelines: (i) Multivariate regression (MR) with 10-fold cross-validation after feature selection based on least absolute shrinkage and selection operator (LASSO), (ii) Random Forest (RF) and (iii) Support Vector Machine (SVM), both with embedded feature selection. All 3 pipelines used as input either the untransformed or the harmonized (with the 4 ComBat versions) radiomic features in combination with the available clinical factors (age, gender, histology, stage, etc.) and included the use of synthetic minority over-sampling technique (SMOTE) to address the data imbalance during training.

**Figure 1:**
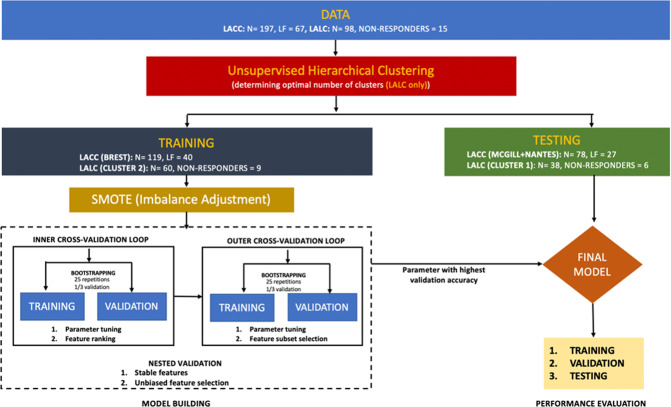
Workflow for the analysis in LACC and LALC datasets.

The overall workflow is illustrated in Fig. 1.

#### Unsupervised hierarchical clustering with silhouette scoring (LALC only)

Using ComBat directly in LALC was not reliable as it would have led to manually define more than 15 labels for 98 patients, based on a very complex and heterogeneous set of acquisition and reconstruction settings across and also within centers (see Supplemental Table 2). Hence, we decided to rely on unsupervised hierarchical clustering to group patients with similar radiomic features distributions,and we also needed an automatically determined optimal number of clusters. In doing this, we assumed that the differences amongst radiomic features due to imaging have higher impact than differences due to different outcomes (*i.e*., responders *vs.* non responders). In order to evaluate the validity of this assumption, we first applied the proposed unsupervised clustering approach to the LACC case where the labels are known and well-defined (*i.e*., the 3 centers Brest, Nantes and McGill). The unsupervised approach correctly identified 3 clusters and was able to assign all patients except one to its correct center (see results section and Supplemental Fig. 4). As there was still a potential risk that the unsupervised clustering would cluster data based on clinical endpoint instead of imaging differences, the resulting clusters were also checked for consistency in terms of their percentage of events (*i.e*., lack of response to TPF).

Hierarchical clustering is a type of unsupervised algorithm which groups data by similarity, *i.e*., it classifies objects without any prior knowledge of the class they belong to, based on the measure of the Euclidean distance^35^. To determine the optimal number of clusters to consider before running the hierarchical clustering, we used ‘silhouette’ scoring, a tool used to validate the clustering. The ‘silhouette’ is then constructed to determine the optimal number of clusters with a ratio scale data (as in the case of Euclidean distance) that is suitable for clearly separated clusters (Supplemental Fig. 1)^36^.

#### Imbalance adjustment

As there were 34% of LF in LACC and 15% of non-responders in LALC, we used SMOTE to facilitate training of the models. SMOTE iteratively performs oversampling of the minority class in order to provide a balanced number of positive and negative cases to the learning algorithm^37^: At every iteration, a new instance of the minority class is synthesized somewhere between one element of the existing minority class and a chosen closest minority class neighbour.

### Machine learning pipelines

#### Multivariate regression with LASSO

The multivariate regression was trained using the features selected by LASSO. LASSO serves as a regularization and variable selection methods for any statistical models^38^. In the case of multivariate cox regression, it penalizes the negative log of the partial likelihood with the LASSO penalty^38^. The algorithm uses a cyclical coordinate descent, which successively optimizes the objective function over a parameter with others kept fixed, and cycles repeatedly until convergence.

#### Random forest

An RF algorithm, making use of an ensemble method for classification consisting of many simple tree classifiers^39^, was also considered. The idea behind ensemble methods is that a team of “weak learners” (single trees) can come together to structure a more suitable learner with a couple of trees creating a forest, which when it is randomized is known as RF.

#### Support vector machine

Finally, a SVM algorithm was included^40^. It works by nonlinearly projecting the training data in the input space to a feature space of higher (infinite) dimension by use of a kernel function. The outcome is a linearly separable dataset that can be separated with the aid of a linear classifier. This undertaking enables the classification of datasets which are usually nonlinearly separable in the input space. In certain instances, classification in high dimension feature spaces results in over-fitting in the input space. Overfitting is controlled through the principle of structural risk minimization^40^.

#### Embedded features selection technique

Both RF and SVM rely on embedded feature selection, which means that the models validation, feature subset selection and hyper parameters optimization steps are performed simultaneously. This technique accommodates feature selection as a section of the model fitting/training process. It is therefore normally specific to a given learning algorithm, *i.e.*, the feature subset selection can be considered as a search in the combined space of feature subsets and hypotheses. For RF, a measure of variable importance estimates the relevance of variables based on a couple of decision trees at the training step^41^. These importance scores from a RF are then used to guide the feature selection process. The feature selection method follows the nested validation approach. This procedure ensures unbiased feature selection and an optimal model less prone to overfitting and selection bias^42^. Starting with an inner validation loop, multiple RF with different bootstrap samples are trained (multiple RF models are fitted for multiple bootstrap samples). Then the variables are ranked using the averaged variables importance across the number of bootstrap iterations, which ensures that more stable features are being selected. After this, an outer validation loop that is a stepwise forward feature selection is being considered to select the number of features that minimize the validation error. As previously described, bootstrap setting is used to assess which subset of features provides the optimal mean prediction error rate. In the case of SVM, the same procedure was considered although, instead of the measure of variable importance as in RF, features are ranked based on the best fine cost of the models and are ranked according to the values of leave-one-out error (*LOO*–*i*), *i.e.*, the feature *i* with the highest value of *LOO*–*i* is ranked first^43^.

#### Final model construction

In order to exploit the feature selection and parameter tuning results, the multiple regression with LASSO, RF and SVM final models were fitted separately with the selected optimal features subset and parameters.

#### Evaluation metrics

Usual performance metrics were computed. Area under the ROC curve (AUC), accuracy, sensitivity and specificity are provided in the Supplemental Material Figs. 5–10. In order to compare the results obtained without and with the different ComBat versions, we decided to focus on two metrics, balanced accuracy (BAcc) and Matthews correlation coefficient (MCC, worst value = −1; best value = +1)^44^. BAcc is calculated as the average of sensitivity and specificity and is an appropriate metric in the presence of strong class imbalance, contrary to standard accuracy. MCC is a reliable metric producing a high score only if the prediction obtained good results in all of the 4 confusion matrices (true positives, false negatives, true negatives and false positives).

### Approval, accordance and informed consent

The study was approved by the local ethics committee of the University Hospital of Brest (references 29BRC19.0006 and 29BCR18.0015 for LALC and LACC respectively). All patients gave their informed consent via a non-opposition form. All procedures were in accordance with the ethical standards of the institutional research committee and with the 1964 Helsinki declaration and its later amendments.

## Results

### Initial analysis

The unsupervised clustering applied to the LACC patient cohort (with known labels) was able to almost perfectly identify patients from the 3 different centers (Supplemental Fig. 4). Only 1 patient from Brest was misclassified and associated with the McGill label.

In the LALC cohort, the unsupervised clustering identified two clusters of 38 and 60 patients, which seems reasonable with respect to the size of the cohort (less than 100 patients), allowing for a sufficiently large number of patients in each label for ComBat estimation (Supplemental Fig. 2). Because both clusters had similar proportion of events (6 non-responders for 38 patients (16%) in cluster 1 and 9 non-responders for 60 patients (15%) in cluster 2), it is very unlikely that this unsupervised differentiation was based on outcome, rather than on differences of features due to center-effect. Subsequently in terms of modelling, cluster 2 was chosen as the reference (for M-ComBat) training set, whereas cluster 1 was chosen as the testing set.

The COV measurements (Table 1) in each of the clinical applications show that: (i). The untransformed data exhibit more variability than their ComBat-harmonized counterparts, and (ii). The data harmonized with the bootstrapped ComBat (B-ComBat) exhibit reduced variability compared to the data harmonized with the non-bootstrapped ComBat. This means that B-ComBat and BM-ComBat have lower levels of dispersion around the mean which should in turn provide more precise and accurate estimates.

**Table 1:** COV computed on the untransformed and four ComBat versions data.

Data	COV	
	LACC	LALC
Untransformed	3039	1987
ComBat	1389	1108
B-ComBat	1385	1082
M-ComBat	1389	1108
BM-ComBat	1385	1082

According to ANOVA, 98% and 96% (in LACC and LALC respectively) of untransformed radiomic features were significantly different between labels at p < 0.01. After harmonization, all of the four ComBat versions completely eliminated significant label related differences across the different cohorts in both datasets, *i.e.*, none of the radiomic features remained significantly different between labels.

**Figure 2:**
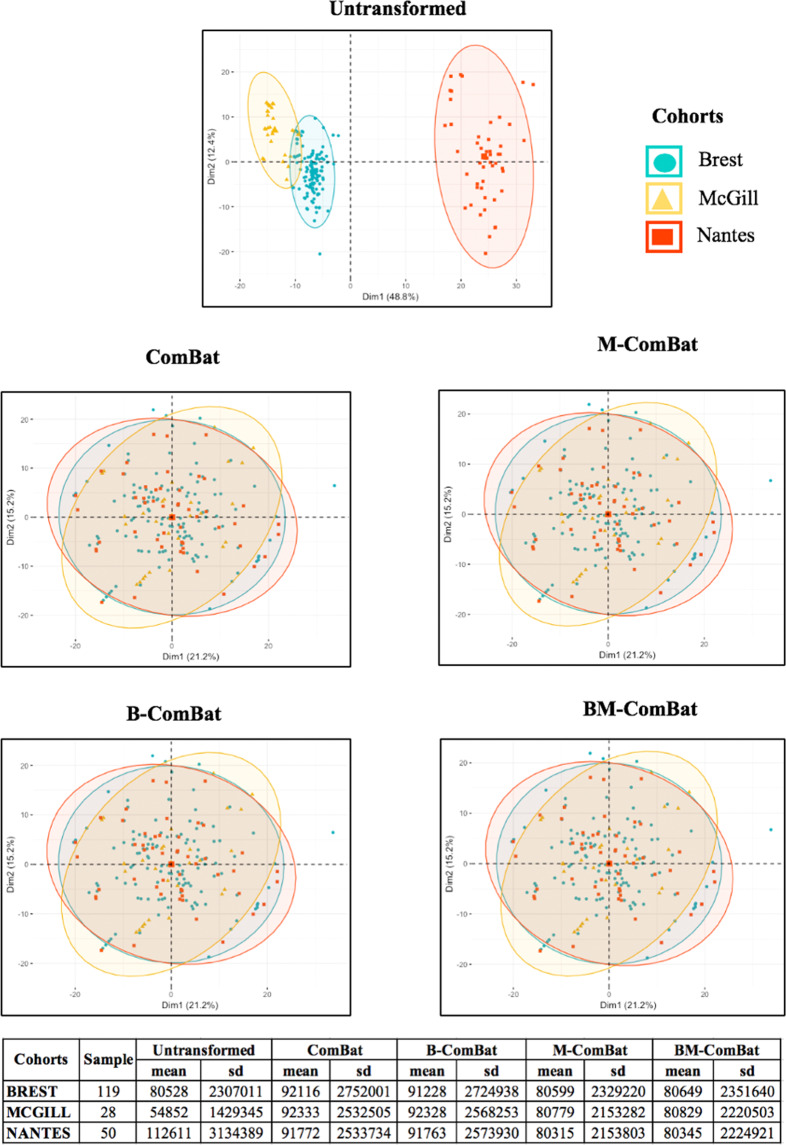
PCA and summary distribution in LACC: Scatter plots of top 2 principal components of the radiomic features across the three labels (centers) using untransformed data or data transformed with the 4 versions of ComBat. (using R (3.5.1) and R Studio (1.1.456, R Studios Inc., Boston, MA, https://cran.rproject. org/).

**Figure 3:**
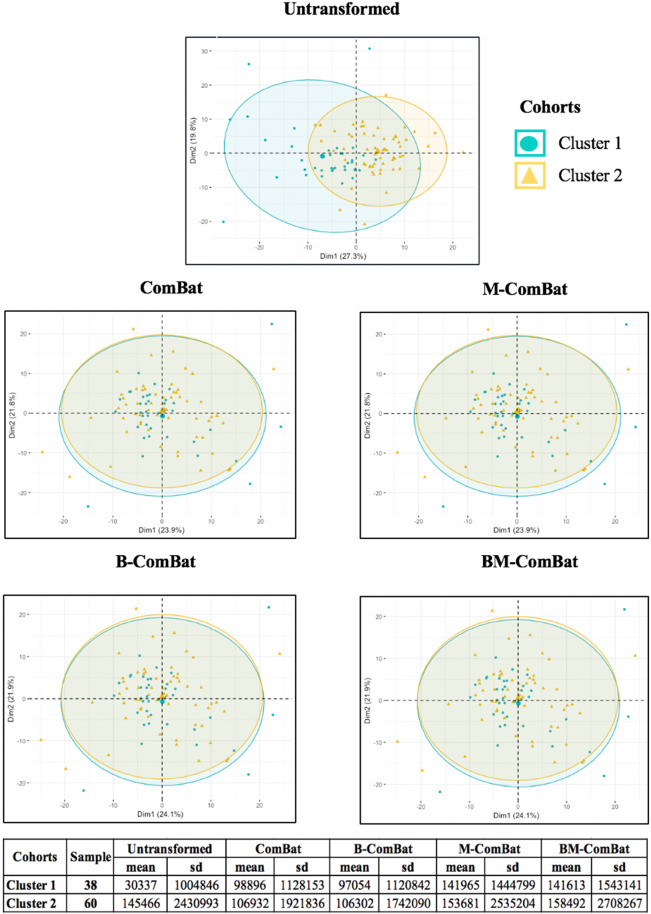
PCA and summary distribution in LALC: Scatter plots of top 2 principal components of theradiomic features across the two labels (clusters) using untransformed data or data transformed with the 4 versions of ComBat (using R (3.5.1) and R Studio (1.1.456, R Studios Inc., Boston, MA, https://cran.rproject. org/).

Scatterplots of the top two principal components of PCA (Figs. 2 and 3, representing 61% and 47% of the information in LACC and LALC, respectively) visually demonstrate the efficiency of all four ComBat versions in removing the differences in radiomic features between labels while shifting the data to different locations (shown by the mean and standard deviation in the tables below the graphs). By transforming all of the data to the global mean rather than a label-specific mean, ComBat (and B-ComBat) alter the location of potential reference samples. On the contrary, transformation by means of M-ComBat (and BM-ComBat), allows data from all labels to overlay each other and at the same time be centered on the chosen reference label (Brest in LACC and cluster 2 in LALC, as shown by the mean and standard deviation in the tables below the graphs). This result clearly demonstrates the interest of the M-ComBat: providing the user with the capability to shift data to a chosen reference standard without losing the capability to correct for individual center effects.

### Predictive modelling using machine learning approaches

Table 2 provides results for the 2 performance evaluation metrics in the testing set, for considering the use of the different ML algorithms in combination with the two clinical datasets, using the 4 versions of ComBat. The same results (including training sets and additional evaluation metrics) are provided in Supplemental Figs. 5–10.

**Table 2:** Performance metrics evaluation of predictive models in LACC and LALC testing sets using the three ML pipelines.

ML	Data	BAcc(%)		MCC [−1,+1]	
		LACC	LALC	LACC	LALC
**MR**	Untransformed	60	27	0.19	−0.39
	ComBat	68	69	0.35	0.31
	B-ComBat	70	71	0.37	0.31
	M-ComBat	68	69	0.35	0.31
	BM-ComBat	70	71	0.35	0.31
**RF**	Untransformed	79	82	0.69	−0.15
	ComBat	84	83	0.72	0.18
	B-ComBat	86	85	0.73	0.23
	M-ComBat	85	84	0.70	0.16
	BM-ComBat	89	86	0.89	0.59
**SVM**	Untransformed	75	41	0.53	−0.13
	ComBat	79	48	0.55	−0.08
	B-ComBat	82	62	0.63	0.18
	M-ComBat	79	58	0.55	0.12
	BM-ComBat	83	60	0.67	0.17

For both patient cohorts, RF provided the best classification accuracy (BAcc of 79-89% in LACC and 82–86% in LALC) compared to MR (60–70% in LACC, 27–71% in LALC) and SVM (75–83% in LACC, 41–62% in LALC).

The absolute increase in performance between the use of the original, untransformed features and the harmonized ones varied depending on the ML algorithms used and between the patient populations considered. For example, a small improvement was seen with RF for LALC (BAcc from 82% to 83%) and a much larger one for MR with LALC (BAcc from 27% to 69%). However, results using harmonized features (whatever version of ComBat was used) were systematically better than using the untransformed ones for all three ML algorithms.

Regarding the two proposed modifications implemented within ComBat, the M modification had a very limited impact on the performance, which was expected since the objective of this modification is simply targeting improved flexibility and not improved performance. It provided very similar (actually most often exactly the same) or slightly better results as the standard ComBat, while allowing for the chosen reference set not to be modified and avoiding some features being transformed into impossible values such as negative volumes.

On the other hand, B-ComBat aimed at improving the harmonization results. Indeed, it provided systematically better (although small in magnitude) performance compared to standard ComBat, in both LACC and LALC and across all ML approaches considered in this study. The comparison between BM-ComBat and B-ComBat led to similar conclusions as with the comparison between M-ComBat and ComBat, with mostly the same or slightly improved performance. All these observations were consistent between the two evaluation metrics (*i.e.*, BAcc and MCC), as well as with the ROC AUC (see Supplemental Figs. 5–10).

## Discussion

In this work, we proposed an improvement of a well-known methodology for eliminating center-effects using radiomic feature based signatures. We have shown the added value of this improvement first using principal component analysis, descriptive statistics and coefficients of variation. Within this context we have shown that the proposed modified ComBat slightly but consistently improved the estimates and the resulting predictive ability of models with all ML algorithms implemented in this work. This improvement was observed across all performance metrics and for both clinical cohorts considered.

All versions of ComBat were able to remove the differences amongst radiomic features caused by systematic imaging effects. The features’ distributions (mean and standard deviation) were all aligned with the chosen reference using M-ComBat and BM-ComBat (Brest for LACC and cluster 2 for LALC). The non-biological variation introduced by differences in imaging systems, acquisition protocols and reconstruction settings led to a dramatic impact on the overall radiomic features distributions as can be seen in the PCA plots (Figs. 2 and 3). These variations are often unavoidable in multicenter studies, as well as in retrospective studies with a long recruitment duration (*e.g.*, when the scanner is replaced by another model at some point). The four ComBat versions were equally effective in eliminating related differences caused by confounding center effects. However, M-ComBat and BM-ComBat were shown to offer a more flexible and robust framework to overcome these differences. The M-modification provides the ability to control the location and scale of the transformed data, whereas the B-modification improves the accuracy of the estimates. Combining the two lead to an improvement in the development and validation of radiomic signatures by combining data from different centers.

Overall, results were consistent for both LACC and LALC cohorts across the 3 different ML approaches in terms of comparison between untransformed and ComBat harmonized datasets, although the absolute performance varied between ML approaches, as already observed in other studies^45^. The performance of the developed models were always improved with any harmonized data compared to untransformed ones (e.g., 60% BAcc in LACC untransformed data compared to 68–70% for harmonized data and 27% BAcc in LALC *vs.* 69–71%, when using MR with LASSO). The absolute difference in performance amongst the 3 ML algorithms can be attributed to the different feature selection techniques^46^. The feature selection techniques embedded within RF and SVM modelling clearly allows to fully span the initial datasets space in order to identify the most relevant combination of features for a given task and are therefore leading to superior results.

The original ComBat algorithm follows a three steps procedure: (i). Data standardization, (ii). Empirical Bayes estimation of prior distribution hyperparameters from standardized data and subsequent estimation of batch effect parameters, which are used in (iii). Correction of batches^17,47^. ComBat has several interesting properties for the purpose of radiomics harmonization: it does not require altering the feature definitions^16,48^ and can therefore be used with any algorithm. It is easy to use and fast. It allows exploiting the entire available information since none of the features are eliminated prior to the analysis^16,48^ and it can be used for both prospective or retrospective data, provided that same disease and corresponding treatment patient cohorts are available in the different centers. ComBat was previously shown to outperform 6 other methods for batch effect removal in microarray datasets from brain RNA samples and two simulated datasets^26^. It was subsequently used successfully in several radiomic studies^9,49^. In contrast, other recent studies exploited basic normalization to achieve the same goal, although with the assumption that while making data more comparable, it does not remove any biological signal of interest^7,22^. A thorough comparison of ComBat with other normalization techniques remains to be carried out specifically in the context of radiomics.

The M- modification provides the user the flexibility to harmonize the features set to a chosen reference, which can be of interest for example if there is a higher confidence or understanding of one dataset compared to the others. The proposed use of bootstrap for initial estimates reduced variances within each center and helped in facilitating bias reduction during center effect parameter estimation by ComBat and M-ComBat respectively and therefore led to an improved center effect removal and predictive performance. Although relatively small, the improvement provided by the use of BM-ComBat was consistent whatever ML algorithm was used for both clinical datasets considering different cancer types and imaging modalities.

Although the proposed modified ComBat provided a consistent improvement in the predictive performance of the developed models in different ML algorithms, we acknowledge the limitations associated with relatively small improvements in combination with small datasets. Our findings will thus require a validation in larger cohorts, as well as in other configurations of numbers of labels. In the case of LALC patient cohort, one limitation is the use of unsupervised hierarchical clustering to automatically identify a likely and reliable number of labels to apply ComBat to. It was especially important to check that the resulting clusters were not defined based on the clinical endpoint but rather systematic differences due to imaging variability: as each resulting clusters had a similar percentage of non-responders, it can be safely assumed that the clusters were indeed obtained mostly based on measured differences due to imaging acquisition and associated processing protocols, rather than different outcome profiles. In addition, when performing the same technique to the LACC datasets with known labels, the unsupervised clustering identified correctly (except for one patient) its correct label (*i.e.*, center). Although our proposed modifications improved the performance of ComBat for harmonization purposes, they do not alleviate some of its inherent limitations. To work properly, ComBat requires available and labelled data in order to perform the estimate and batch correction. In addition, when new datasets are added, they have to be combined with the other available ones and the harmonization has to be re-established on the entire database. Similarly, in order to apply a developed/validated model (*i.e.*, a combination of harmonized radiomic features with an associated threshold value) to a new patient from another center not previously included, there is currently no direct method to apply the previously determined harmonization transform to the radiomic features values of this new patient in order to determine his/her prediction. Our future work will investigate how to address these remaining limitations.

Finally, in the present work we considered the entire set of radiomic features irrespectively of their robustness. An alternative strategy consists in identifying features robust to changes in acquisition and reconstruction settings prior to feeding them to the machine learning pipeline. Such a feature selection procedure can help building more robust models, potentially without the need for harmonization, since only features insensitive to multicenter variability are exploited. However, it suffers from a potential loss of information, as features identified as unreliable are usually discarded before being evaluated and the most robust/reproducible features might not necessarily be the most discriminant. In addition, the size of the radiomic features set would depend on the chosen threshold of what is considered robust enough. A full comparison between the two approaches will be carried out in our future work.

## Conclusion

The hybrid bootstrapped ComBat (B-ComBat and BM-ComBat) versions are modifications to a well-established methodology allowing a more reliable estimation (B) and ability to control the location and scale of center-effect adjusted data (M). It was shown to slightly but consistently improve the performance of predictive radiomics models in a multicenter context, whatever machine learning technique was used. We thus recommend the use of this BM-modified ComBat approach for the future development and validation of predictive models in a multicenter context.

## Supplementary information

Supplementary Information.

## Data Availability

Radiomic features can be made available on request for specific research purposes.
