# Supplementary material for: Performance comparison of modified ComBat for harmonization of radiomic features for multicenter studies
Source: Sci Rep. 2020 Jun 24;10:10248. doi: 10.1038/s41598-020-66110-w (PMC7314795; doi:10.1038/s41598-020-66110-w)

Performance comparison of modified ComBat for harmonization of radiomic features for multicentric studies

Ronrick Da-ano[1], Ingrid Masson[1,2], François Lucia[1,3], Mélanie Doré[2], Philippe Robin[4], Joanne Alfieri[5], Caroline Rousseau[6,11], Augustin Mervoyer[2], Caroline Reinhold[7], Joel Castelli [8,9], Renaud De Crevoisier[8,9], Jean-François Ramée[10], Olivier Pradier[1,3], Ulrike Schick[1,3], Dimitris Visvikis[1*], Mathieu Hatt[1*]

[*] equally contributed

[1]INSERM, UMR 1101, LaTIM, University of Brest, Brest, France,
[2]Department of Radiation Oncology, Institut de cancérologie de l'Ouest René-Gauducheau, Saint-Herblain, France
[3]Radiation Oncology Department, University Hospital, Brest, France,
4 Department of Nuclear Medicine, University of Brest, Brest, France
[5] Department of Radiation Oncology, McGill University Health Centre, Montreal, Quebec
[6]Department of Nuclear Medicine, Institut de cancérologie de l'Ouest René-Gauducheau, Saint-Herblain, France
[7]Department of Radiology, McGill University Health Centre, Montreal, Canada,
[8]Radiotherapy Department Cancer, Institute Eugène Marquis, Rennes, France
[9]University of Rennes 1, LTSI, Rennes, France
[10]Department of Medical Oncology, Centre Hospitalier de Vendée, La Roche sur Yon, France
[11]CRCINA, University of Nantes, INSERM UMR1232, CNRS-ERL6001, Nantes, France

Corresponding author:
Ronrick Da-ano, PhD candidate
LaTIM, INSERM, UMR 1101,
IBRBS (Institut Brestois de la Recherche en Biologie-Santé)
22 rue Camille Desmoulins, 29238 Brest, France
ronrickarnaiz@gmail.com

Competing Interests statement:
The authors declare no competing interests.

Approval, accordance and informed consent:
The study was approved by the local ethics committee of the University Hospital of Brest (references 29BRC19.0006 and 29BCR18.0015 for LALC and LACC respectively). All patients gave their written consent via a non-opposition form. All procedures were in accordance with the ethical standards of the institutional research committee and with the 1964 Helsinki declaration and its later amendments.

Authors contributions statement:
M. H., D. V. and R. D. designed the study.
R. D. developed the methods and performed the statistical analysis.
U. S., I. M. and F. L. collected and processed the images and calculated the features.
M. D., P. R., J. A., C. R., A. M., C. R., J. C., R. DC., J.F. R., and O. P. contributed to writing and reviewed/approved the manuscript.

Funding:
This study was partly funded by 766276 PREDICT H2020-MSCA-ITN-2017 and M2R201806006004

Data availability statement:
Radiomic features can be made available on request for specific research purposes.
Supplementary materials

Supplementary table 1: PET/CT and MRI protocols in Brest (A), Nantes (B), and McGill (C)

(A)

	Preparation	Technical characteristics
PET	Patients fasted for 4h before acquisition<br>The blood glucose level had to be less than 7 mmol/L<br>Injection of 5 MBq/kg of ^{18}F-FDG<br>PET acquisitions were carried out approximately 60min after injection	Routine clinical image reconstruction protocols were used: for the Philips GEMINI, data were reconstructed using the RAMLA 3D (2 iterations, relaxation parameter 0.05) whereas for the Siemens Biograph, images were reconstructed with Fourier rebinning (FORE) followed by OSEM (2 iterations, 8 subsets). In both cases images were corrected for attenuation using the corresponding CT, reconstructed with a 2×2×2 mm^3 voxels grid and post-filtered with a 5-mm FWHM 3D Gaussian.
CT	N/A (acquired with the PET/CT acquisition)	The CT consisted of a 64-slice multidetector-row spiral scanner with a transverse field of view of 700 mm. Standard CT parameters were used: a collimation of 16×1.2 mm^2, pitch 1, tube voltage of 120 kV, and effective tube current of 80 mA.

Sequences	Plane	Technical characteristics
T2-w	Axial (renal hilum-pubis)	Axial: TR=3425ms, TE=110ms, NSA: 2, ST/G: 4.5/1, matrix: 340×350, FOV: 38, AT=3.30 min
	Sagittal	Sagittal: TR=3425ms, TE=110ms NSA: 3, ST/G: 3.5/1.2, matrix: 348×276, FOV: 25, AT=3.36 min
	Axial oblique (perpendicular to cervical axis or/and along with endometrial cavity axis)	Axial oblique: TR=3425ms, TE=110ms, NSA: 6, ST/G: 4/0.4, matrix: 256/176, FOV: 18, AT=3.30 min
T1-w	Axial (renal hilum-pubis)	TR=575ms, TE=7.7 to 17ms, NSA: 1, ST/G: 6/2, matrix: 300×205, FOV: 36, AT=2.16 min
T1-FS+CE	Axial and sagittal<br><br>All except two allergic patients (training set) received a 0.1mmol/kg injection of	TR=540ms, TE=10 to 12ms, NSA: 2, ST/G: 4.5/1, matrix: 360×252, FOV: 38, AT=3.28 min

	gadobenate dimeglumine (Multihance; Bracco Diagnostics, Milan, Italy).	
DWI	Axial oblique and sagittal, b value=(0, 400, 1000) s/mm²<br>ADC maps creation: For each acquisition, the ADC was computed voxel by voxel as the slope of the linear regression of the logarithm of the DWI exponential signal decay on the three b-values.	TR=3900ms, TE=80ms NSA: 12, ST/G: 6/0 matrix: 124×174, FOV: 35, AT=3.40 min

Abbreviations: T2-W: T2-weighted, T1-W: T1-weighted, T1-FS+CE: T1 fat-suppressed with contrast enhancement, DWI: diffusion-weighted imaging,
AT: acquisition time, TR: repetition time, TE: echo time, NSA: number of signal acquisition, ST (mm): slice thickness, G (mm): gap, FOV (cm): field of view (right to left).

(B)

	Preparation	Technical characteristics
PET	Patients fasted for 4h before acquisition<br>The blood glucose level had to be less than 7 mmol/L<br>Injection of 5 MBq/kg of ^{18}F-FDG<br>PET acquisitions were carried out approximately 60min after injection	Routine clinical image reconstruction protocols were used: for the Siemens Biograph, images were reconstructed with Fourier rebinning (FORE) followed by OSEM (2 iterations, 8 subsets). Images were corrected for attenuation using the corresponding CT, reconstructed with a 2×2×2 mm^3 voxels grid and post-filtered with a 5-mm FWHM 3D Gaussian.
CT	N/A (acquired with the PET/CT acquisition)	The CT consisted of a 64-slice multidetector-row spiral scanner with a transverse field of view of 700 mm. Standard CT parameters were used: a collimation of 16×1.2 mm^2, pitch 1, tube voltage of 120 kV, and effective tube current of 80 mA.

Sequences	Plane	Technical characteristics
T2-w	Axial (renal hilum-pubis)	Axial: TR=3445ms, TE=105ms, NSA: 2, ST/G: 4.5/1, matrix: 384×384, FOV: 38, AT=3.25 min

	Sagittal	Sagittal: TR=3425ms, TE=110ms NSA: 3, ST/G: 3.5/1.2, matrix: 348×276, FOV: 25, AT=3.25 min
	Axial oblique (perpendicular to cervical axis or/and along with endometrial cavity axis)	Axial oblique: TR=3445ms, TE=105ms, NSA: 6, ST/G: 4/0.4, matrix: 276x194, FOV: 18, AT=3.25 min
T1-w	Axial (renal hilum-pubis)	TR=575ms, TE=8.5 to 16ms, NSA: 1, ST/G: 6/2, matrix: 320×240, FOV: 36, AT=2.16 min
T1-FS+CE	Axial and sagittal<br>All except two allergic patients (training set) received a 0.1mmol/kg injection of gadolinium chelate (gadoteric acid; Dotarem Guerbet, Aulnay-sous-Bois, France).	TR=560ms, TE=10 to 12ms, NSA: 2, ST/G: 4.5/1, matrix: 320×240, FOV: 38, AT=3.22 min
DWI	Axial oblique and sagittal, b value=(0, 400, 1000) s/mm²<br>ADC maps creation: For each acquisition, the ADC was computed voxel by voxel as the slope of the linear regression of the logarithm of the DWI exponential signal decay on the three b-values.	TR=3800ms, TE=80ms NSA: 12, ST/G: 6/0 matrix: 148×192, FOV: 35, AT=3.30 min

Protocole 1
Abbreviations: T2-W: T2-weighted, T1-W: T1-weighted, T1-FS+CE: T1 fat-suppressed with contrast enhancement, DWI: diffusion-weighted imaging,
AT: acquisition time, TR: repetition time, TE: echo time, NSA: number of signal acquisition, ST (mm): slice thickness, G (mm): gap, FOV (cm): field of view (right to left).

Sequences	Plane	Technical characteristics
T2-w	Axial (renal hilum-pubis)	Axial: TR=3435ms, TE=105ms, NSA: 2, ST/G: 4.5/1, matrix: 384×384, FOV: 38, AT=3.30 min
	Sagittal	Sagittal: TR=3435ms, TE=110ms NSA: 3, ST/G: 3.5/1.2, matrix: 348×276, FOV: 25, AT=3.35 min
	Axial oblique (perpendicular to cervical axis or/and along with endometrial cavity axis)	Axial oblique: TR=3435ms, TE=105ms, NSA: 6, ST/G: 4/0.4, matrix: 256x194, FOV: 18, AT=3.25 min
T1-w	Axial (renal hilum-pubis)	TR=575ms, TE=9 to 15ms, NSA: 1, ST/G: 6/2, matrix: 320×240, FOV: 36, AT=2.16 min

	Axial and sagittal	
T1-FS+CE	All except two allergic patients (training set) received a 0.1mmol/kg injection of gadolinium chelate (gadoteric acid; Dotarem Guerbet, Aulnay-sous-Bois, France).	TR=550ms, TE=10 to 12ms, NSA: 2, ST/G: 4.5/1, matrix: 320×256, FOV: 38, AT=3.22 min
DWI	Axial oblique and sagittal, b value=(0, 400, 1000) s/mm² ADC maps creation: For each acquisition, the ADC was computed voxel by voxel as the slope of the linear regression of the logarithm of the DWI exponential signal decay on the three b-values.	TR=3750ms, TE=80ms NSA: 12, ST/G: 6/0 matrix: 156×196, FOV: 35, AT=3.30 min

Protocole 2
Abbreviations: T2-W: T2-weighted, T1-W: T1-weighted, T1-FS+CE: T1 fat-suppressed with contrast enhancement, DWI: diffusion-weighted imaging,
AT: acquisition time, TR: repetition time, TE: echo time, NSA: number of signal acquisition, ST (mm): slice thickness, G (mm): gap, FOV (cm): field of view (right to left).

(C)

	Preparation	Technical characteristics
PET	Patients fasted for 4h before acquisition The blood glucose level had to be less than 7 mmol/L Injection of 8.14 MBq/kg of ^{18}F-FDG PET acquisitions were carried out approximately 60min after injection	Routine clinical image reconstruction protocols were used: for the Discovery ST, images were reconstructed with OSEM (2 iterations, 8 subsets). Images were corrected for attenuation using the corresponding CT, reconstructed with a 3.65×3.65×3.27 mm^3 voxels grid and post-filtered with a 5-mm FWHM 3D Gaussian.
CT	N/A (acquired with the PET/CT acquisition)	The CT consisted of a 16-slice multidetector-row spiral scanner with a transverse field of view of 700 mm. Standard CT parameters were used: a collimation of 16×1.2 mm^2, pitch 1, tube voltage of 140 kV, and effective tube current of 90 mA.

Sequences	Plane	Technical characteristics
T2-w	Axial (renal hilum-pubis)	Axial: TR=4575ms, TE=100ms, NSA: 4, ST/G: 4/0, matrix: 512×256, FOV: 24, AT=3.25 min
	Sagittal	Sagittal: TR=4575ms, TE=100ms, NSA: 4, ST/G: 4/0, matrix: 512×256, FOV: 24, AT=3.25 min
	Axial oblique (perpendicular to cervical axis or/and along with endometrial cavity axis)	Axial oblique: TR=4000ms, TE=100ms, NSA: 4, ST/G: 4/0, matrix: 512×256, FOV: 24, AT=3.25 min
T1-w	Axial (renal hilum-pubis)	TR=565ms, TE=9 to 11ms, NSA: 1, ST/G: 4/0, matrix: 320×192, FOV: 26, AT=3.26 min
T1-FS+CE	Axial and sagittal<br>All received a 0.1mmol/kg injection of gadolinium chelate (Gadovist; Bayer, Canada).	TR=3.6ms, TE=1.75ms, NSA: 1, ST/G: 4/0, matrix: 320×192, FOV: 26, AT=3.26 min
DWI	Axial oblique and sagittal, b value=(0, 500, 1000) s/mm²<br>ADC maps creation: For each acquisition, the ADC was computed voxel by voxel as the slope of the linear regression of the logarithm of the DWI exponential signal decay on the three b-values.	TR=5000ms, TE=69ms NSA: 8, ST/G: 6/0 matrix: 128×256, FOV: 32, AT=3.35 min

Abbreviations: T2-W: T2-weighted, T1-W: T1-weighted, T1-FS+CE: T1 fat-suppressed with contrast enhancement, DWI: diffusion-weighted imaging,
AT: acquisition time, TR: repetition time, TE: echo time, NSA: number of signal acquisition, ST (mm): slice thickness, G (mm): gap, FOV (cm): field of view (right to left).

Supplemental Table2 - Acquisition and reconstruction characteristics for CT imaging of patients included (LALC)
Scan type was helical for all considered models.

Manufacturer /Model	CT slices	detector configuration (mm)	slice thickness (mm)	tube voltage (kV)	sFOV (mm)	e×posure time (msec)	tube current (mA)	e×posure (mAs)	spiral pitch factor	reconstruction FOV	reconstruction interval	reconstruction matri×	reconstruction kernel
GE/LightSpeed VCT	64	32 × 0.625 or 64 × 0.625	0.625 or 1.25 or 2	120	320	500-1277	80-1095	2-530	0.53 or 1	183-267	0.625 or 1	512 × 512	B30s or STANDARD or SOFT or DETAIL
GE/Brightspeed	11	16 × 1.25 or 16 × 0.625	0.625 or 1.25	120	250	961-1191	69-376	mai-19	0.93 or 0.5625	190-254	0.625 or 0.7 or 5	512 × 512	DETAIL or SOFT or STANDARD
Siemens/SOMATOM Definition AS	64	20 × 0.6 or 32 × 0.6 or 16 × 1.2	1 or 3	100	500	439-1000	44-229	44-286	0.8 or 1	221-500		512 × 512	B30s or I30s or I31f or I50s or I70h
Philips/Ingenuity CT	64	64 × 0.625	0.9 or 1.5	120	500	367-714	215-633	119-3970	0.9923 to 1.3	223-361	-0.75 to 0.8	512 × 512	C or B or SOFT TISSUE
Siemens/Sensation 16	16	16 × 0.75 or 16 × 0.6	0.75 or 1 or 1.5 or 2	120	500	750	101-161	101-200		170-500		512 × 512	B30s or B31s
Siemens/SOMATOM Definition AS+	64	64 × 0.6	1 or 1.5	120	500	1000	116-212	165-265	0.7	235-289		512 × 512	B31s or B40s or I31s
Philips/Brilliance 40	40	40 × 0.625	1.4 or 1.5	120	500		225-462	250		200	-1 to 0.75	512 × 512	B or C
Siemens/Sensation 64	64	64 × 0.6 z-FFS (32 × 0.6)	1 or 2	120	500	1000	198	220		241		512 × 512 or 1024 × 1024	B31s or B41s
Siemens/Emotion 16	16	16 × 0.6	0.75 or 1 or 3	130	500	1000	56-345	74-230		210-216		512 × 512	B30s or B41s
Philips/Brilliance 16	16	16 × 0.75	1 or 1.5	120	177-230		240	200		177-230	0.5 or 0.8	512 × 512	C
GE/Optima CT520	16	16 × 0.625	0.625	120	500	1099	346	18	0.93	271	0.625	512 × 512	DETAIL
Hitachi/Scenaria CT	64	64× 0.625	1.25	120	240	500	594	297		240		512 × 512	83
GE/Optima CT540	16	16 × 0.625	1.25	100	500	1042	194	18	1	263	1 or 0.5	512 × 512	DETAIL
Siemens/Sensation Open			1.5	120	500	1000	197-217	218-241		168-208		512 × 512	B30s or B31s
GE/Discovery CT750 HD	64	64 × 0.625	0.625	120	320	1095	150	2	0.52	270	0.625	512 × 512	DETAIL
Toshiba/Aquilion PRIME	40	40 × 1	1	120	320	500	100	50	0.825	242		512 × 512	FC08
GE/BrightSpeed Q×/i	4	16 × 0.625	0.625	120	250	961	102	4	0.93	250	-0.625	512 × 512	SOFT

GE/LightSpeed Pro 16	16	32 × 0.625	1.25	120	250	1191	197	21	0.93	219		512 × 512	STANDARD
GE/Optima CT660	64	32 × 0.625	0.625	120	320	800	200	5	0.97	246	0.625	512 × 512	STANDARD
Siemens/Biograph 64	64	16 × 1.2	3	120	500	1000	41	41	1	500		512 × 512	I30s
Siemens/Biograph 20	20	16 × 1.2	1.5	140	500	500	53	33	0.8	300		512 × 512	I30f
GE/Discovery ST	8	4 × 2.5	2.5	120	500	819	120	16	1.5	700		512 × 512	STANDARD
Philips/Ingenuity Fle×	32		1.5	120	500	1332	277	369		164	0.75	512 × 512	B
Siemens/SOMATOM Definition Edge	64	64 × 0.6	1	80	500	285	316	75	1.2	354		512 × 512	I30f
Philips/Brilliance 10	10		1.5	120	500		165	248				512 × 512	C
GE/LightSpeed Ultra	8	8 × 1.25	1.25	140	250	1546	381	41	0.625			512 × 512	STANDARD

Supplemental table 3 - list of radiomic features extracted from CE-CT
Details of the definitions and computation of the radiomic features are available in the IBSI reference document:
https://ibsi.readthedocs.io/en/latest/04_Image_features.html

Category	order	Type	Features
Shape			Volume, Approximate Volume, Surface area, Surface to volume ratio, Compactness 1, Compactness 2, Spherical disproportion, Sphericity, Asphericity, Max 3D diameter, Major axis length, Minor axis length, Least axis length, FlatnessElongation
Intensity	first	Intensity histogram features	Mean, Max, Min, Variance, Standard Deviation, Skewness, Kurtosis, Energy, Entropy, Area under the IVH curve, SUV
Texture	second	Grey-level Cooccurrence Matrix (GLCM)	Max, Average, Variance, Entropy, Difference average, Difference Variance, Difference Entropy, Sum Average, Sum Variance, Sum Entropy, Angular Second Moment, Inverse Difference, Inverse Difference normalised, Inverse Difference moment, Inverse Difference moment normalised, Inverse variance, Correlation, Autocorrelation, Cluster tendency, Cluster Shade, Cluster prominence, Information correlation first, Information correlation second
	second	Neighbourhood greytone difference matrix (NGTDM)	Complexity, Busyness, Contrast, Coarseness, Texture strength
	third	Grey-level Run-Length matrix (GLRLM)	Short runs emphasis, Long runs emphasis, Grey level non uniformity, Run length non-uniformity, Run percentage, Low grey level run emphasis, High grey level run emphasis, Short run low grey level emphasis, Short run high grey level emphasis, Long run low grey level emphasis, Long run high grey level emphasis, Normalised grey level non-uniformity, Normalised run length non-uniformity, Grey level variance, Run length variance, Run entropy
	third	Grey-level Size Zone Matrix (GLSZM)	Small Zone Emphasis, Large Zone Emphasis, Low grey level zone emphasis, High grey level zone emphasis, Small zone low grey level emphasis, Small zone high grey level emphasis, Large zone low grey level emphasis, Large zone high grey level emphasis, Grey level non-uniformity, Normalised grey level non-uniformity, Zone size non-uniformity, Normalised zone size non-uniformity, Zone percentage, Grey level variance, Zone size variance, Zone size entropy

Supplemental Figure 1- Graph in determining the optimal number of clusters based on Silhouette scoring in LALC data .*(using R (3.5.1) and R Studio (1.1.456,R Studios Inc., Boston,MA)* https://cran.r-project.org/).

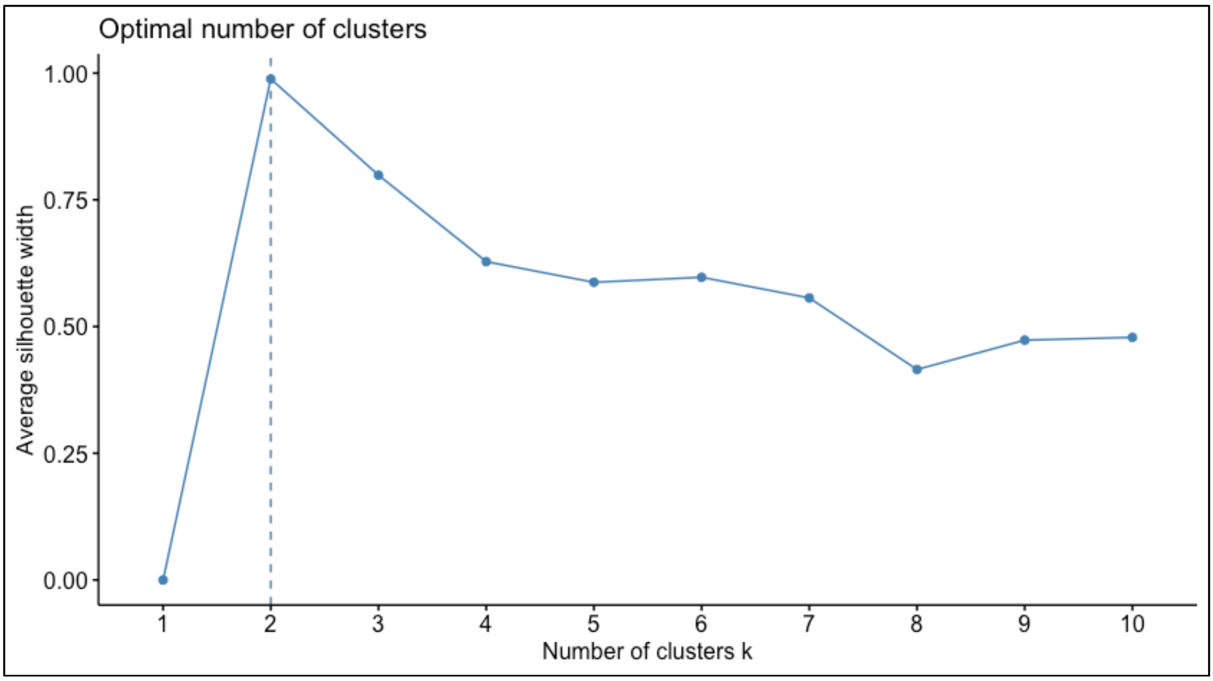

Supplemental Figure 2 - Identifying number of patients based on separated clusters in LALC data .*(using R (3.5.1) and R Studio (1.1.456,R Studios Inc., Boston,MA)* https://cran.r-project.org/).

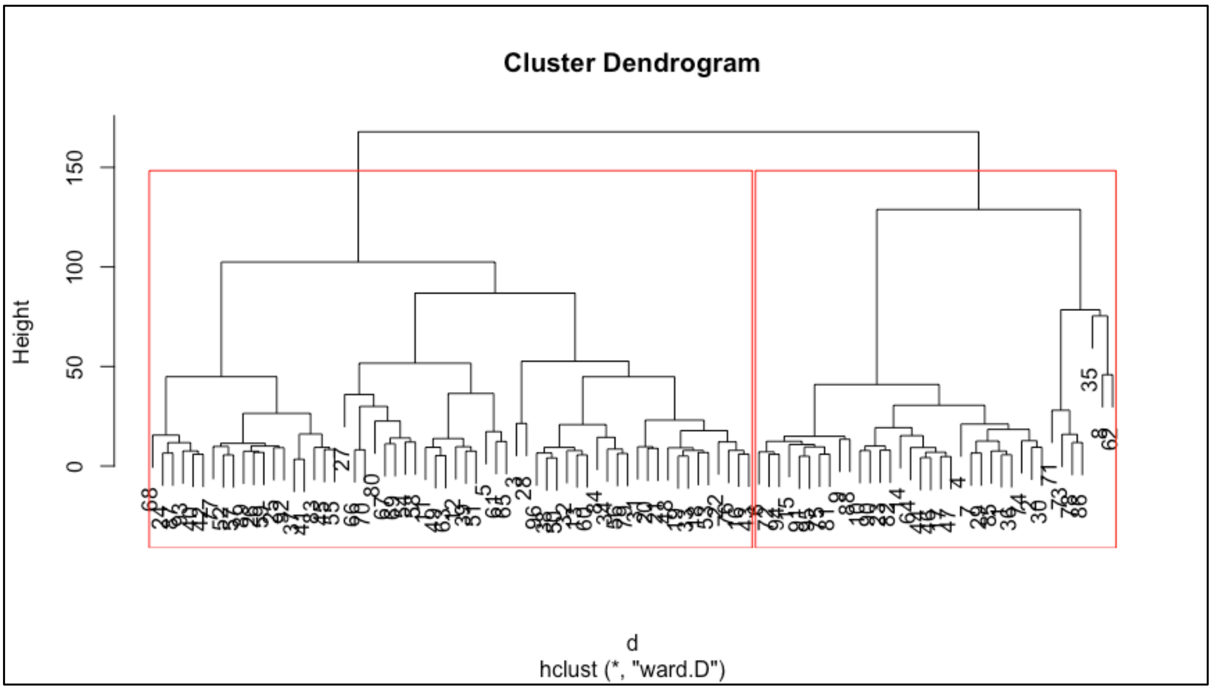

Supplemental Figure 3 - Graph in determining the optimal number of clusters based on Silhouette scoring in LACC data *.(using R (3.5.1) and R Studio (1.1.456,R Studios Inc., Boston,MA)* https://cran.r-project.org/).

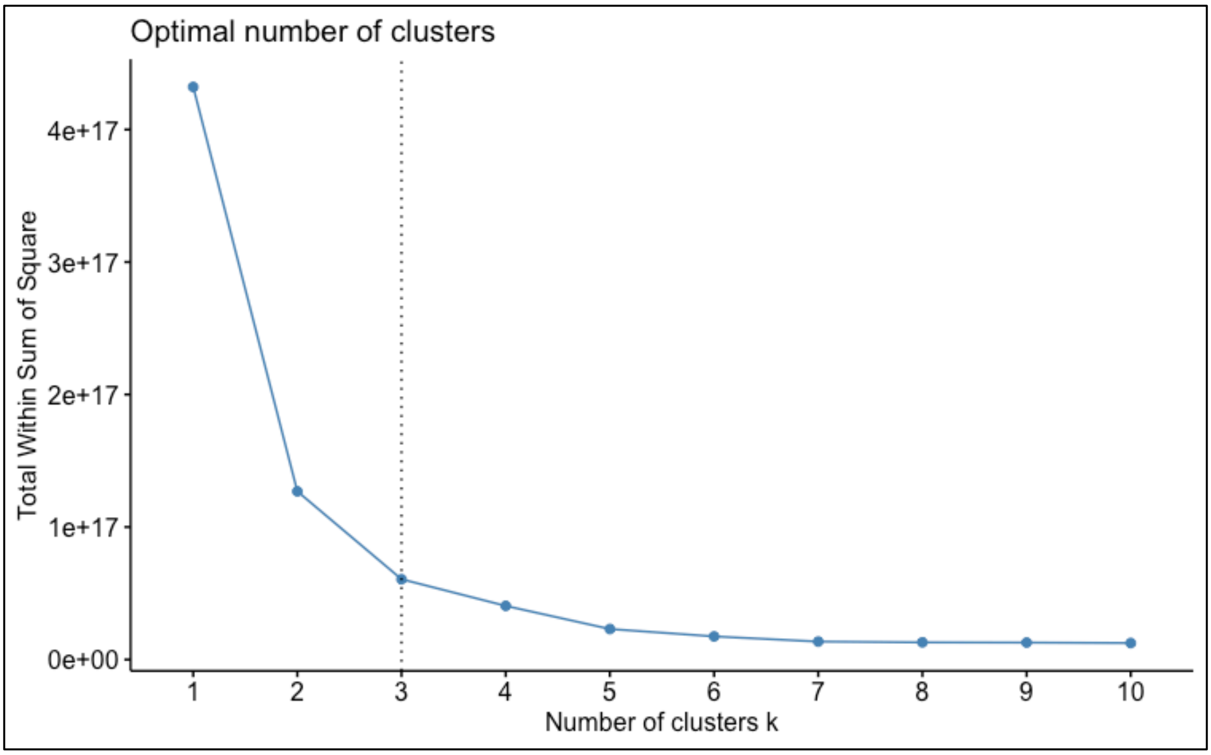

Supplemental Figure 4 - Identifying number of patients based on separated clusters in LACC data *(using R (3.5.1) and R Studio (1.1.456,R Studios Inc., Boston,MA)* https://cran.r-project.org/).

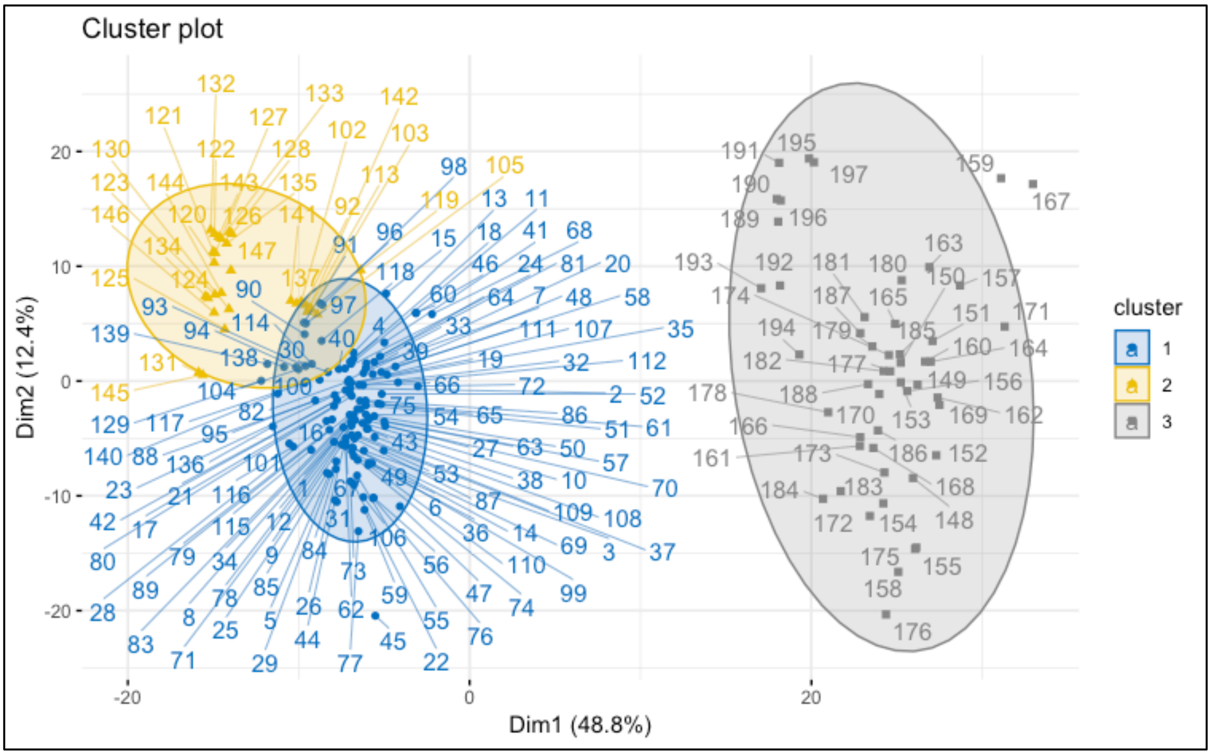

Supplemental Figure 5 - Performance metrics evaluation of predictive models in LACC data using MR with LASSO.

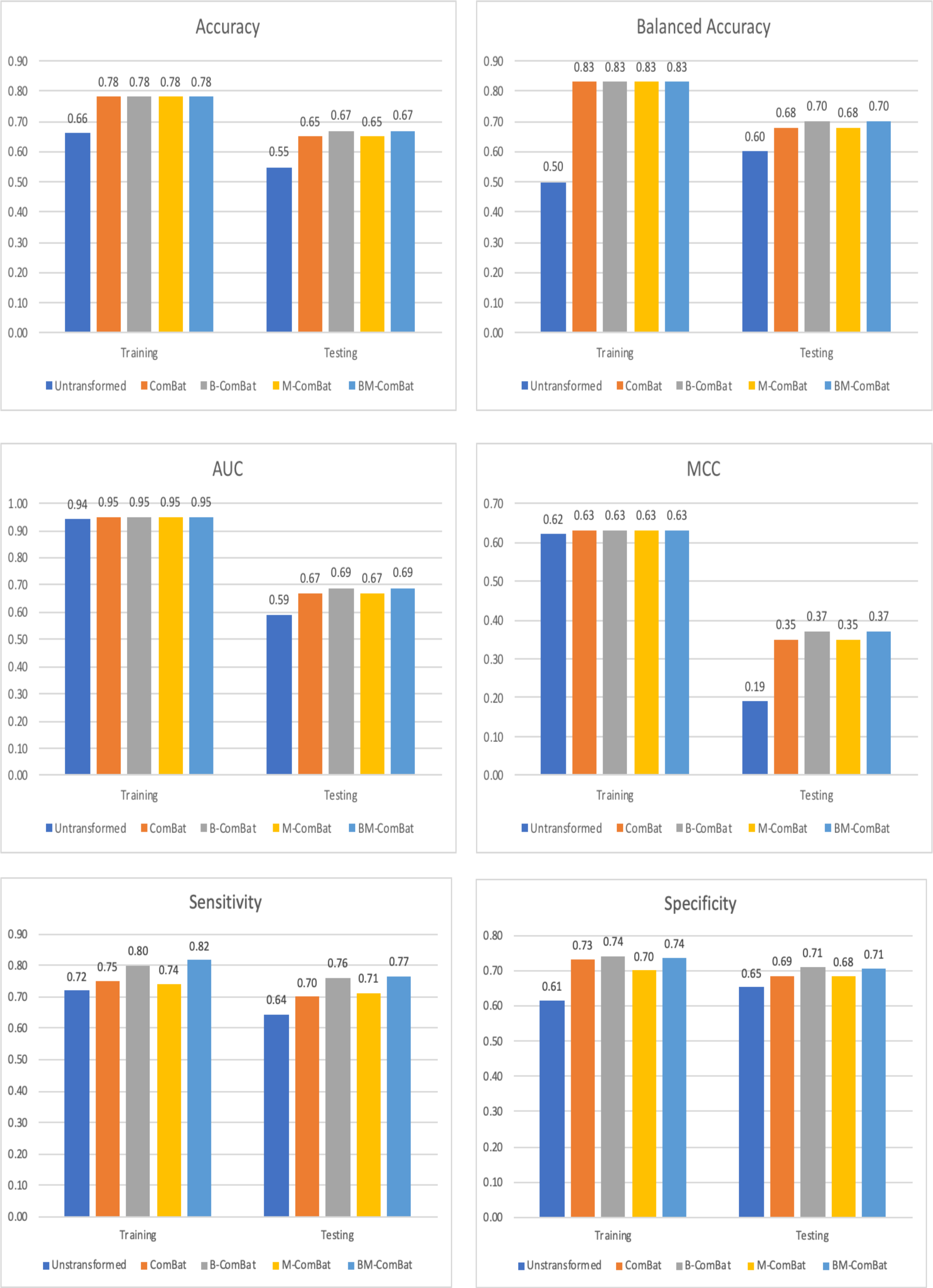

Supplemental Figure 6 - Performance metrics evaluation of predictive models in LALC data using MR with LASSO.

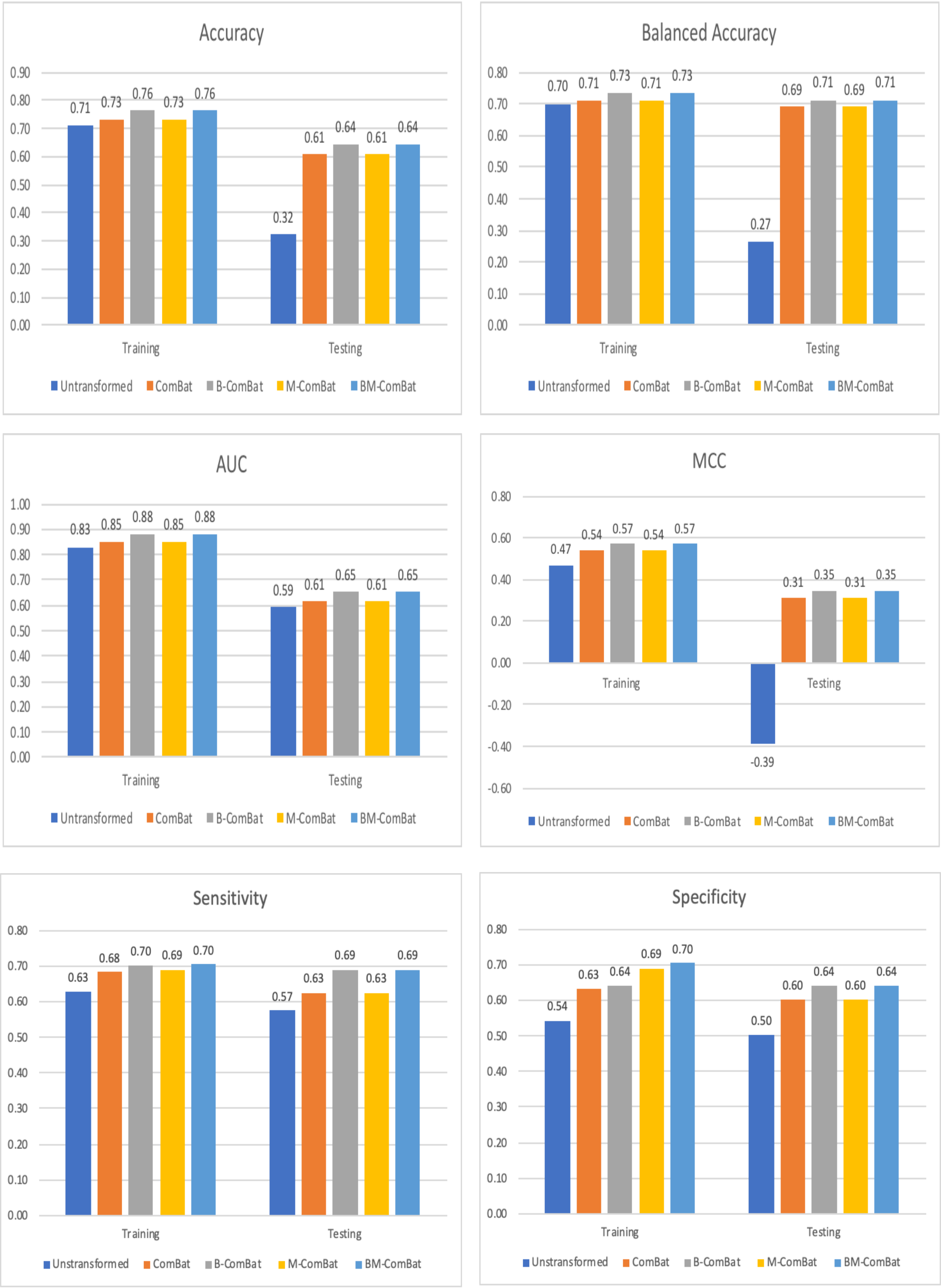

Supplemental Figure 7 - Performance metrics evaluation of predictive models in LACC data using RF.

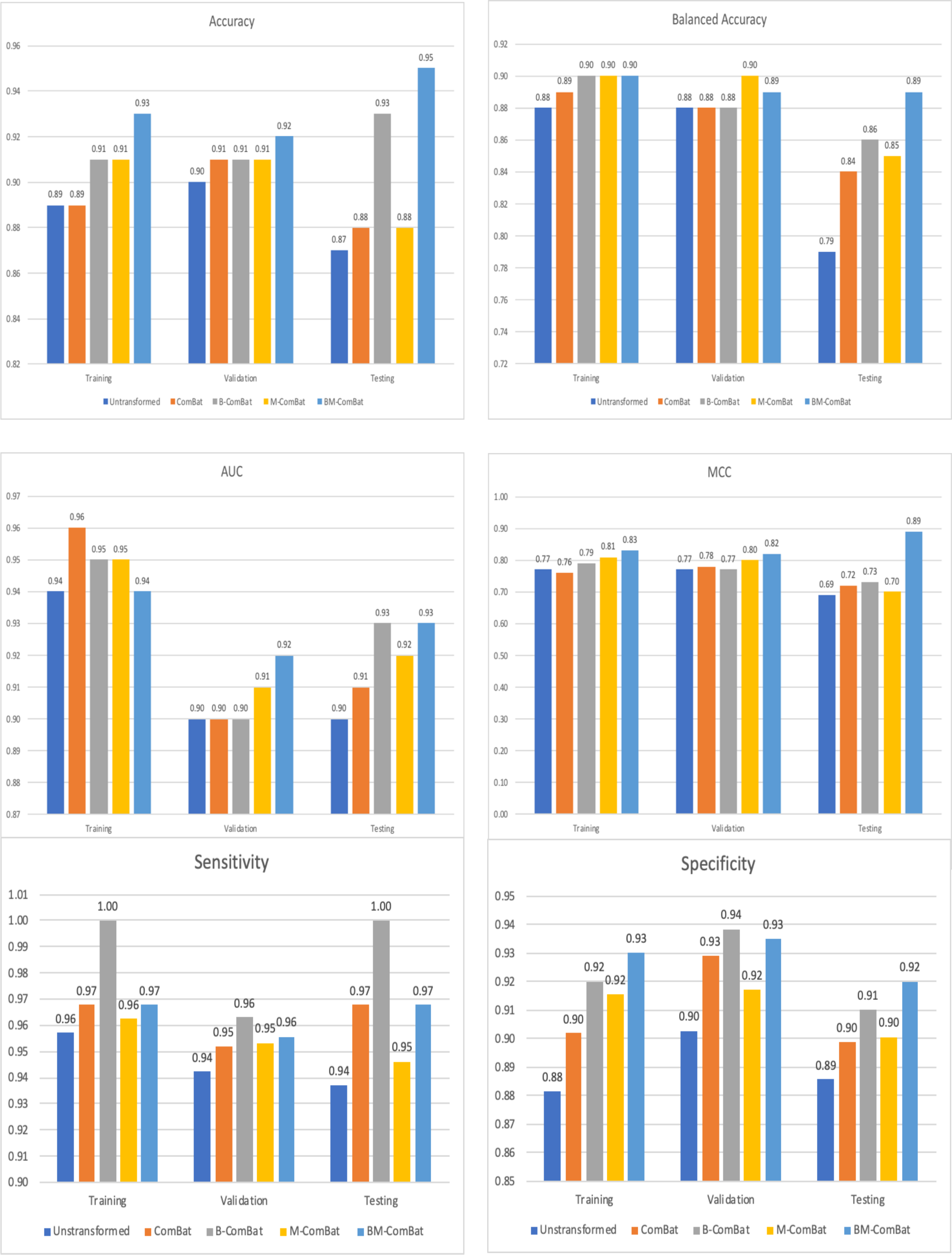

Supplemental Figure 8 - Performance metrics evaluation of predictive models in LALC data using RF.

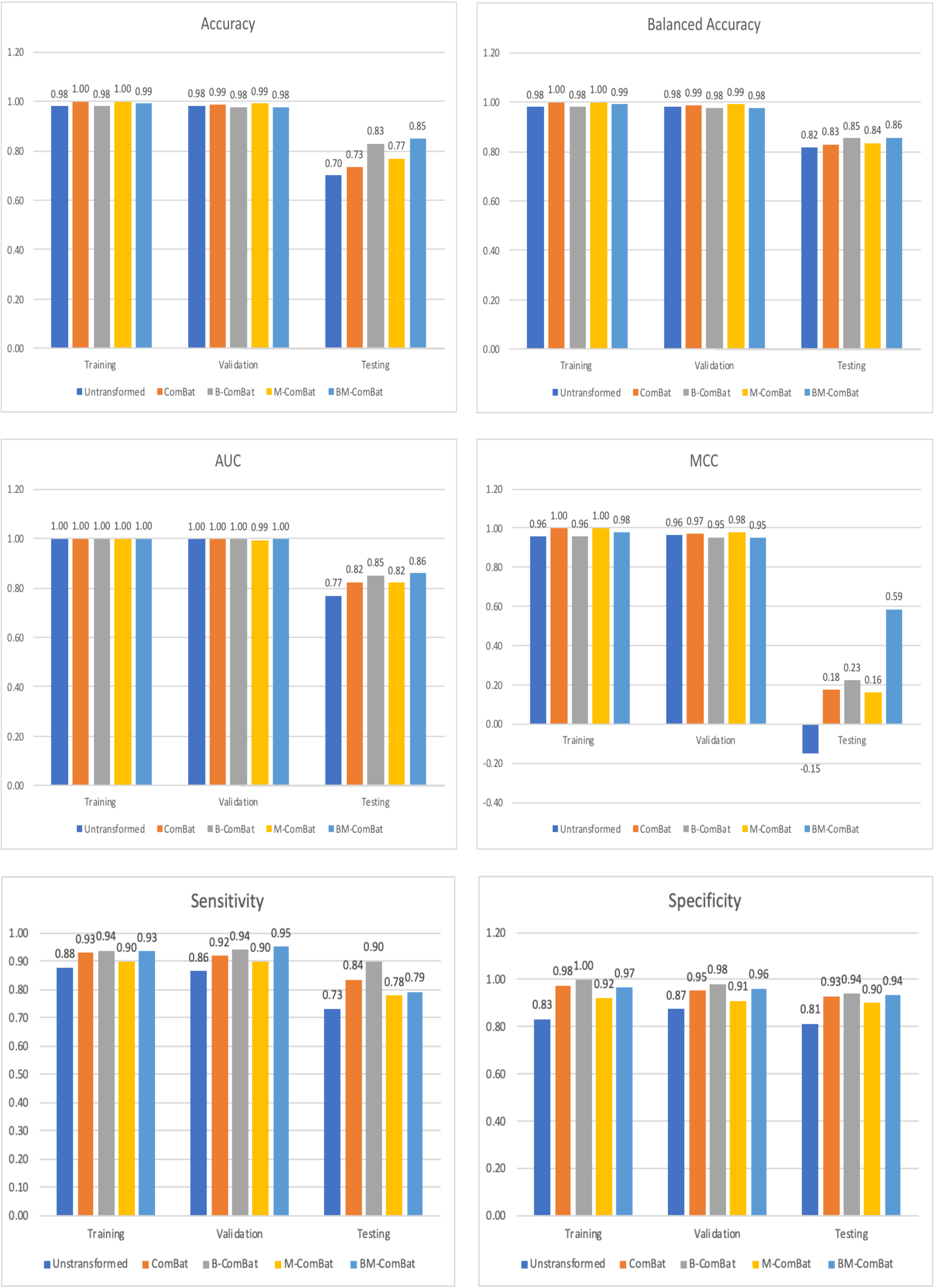

Supplemental Figure 9 - Performance metrics evaluation of predictive models in LACC data using SVM.

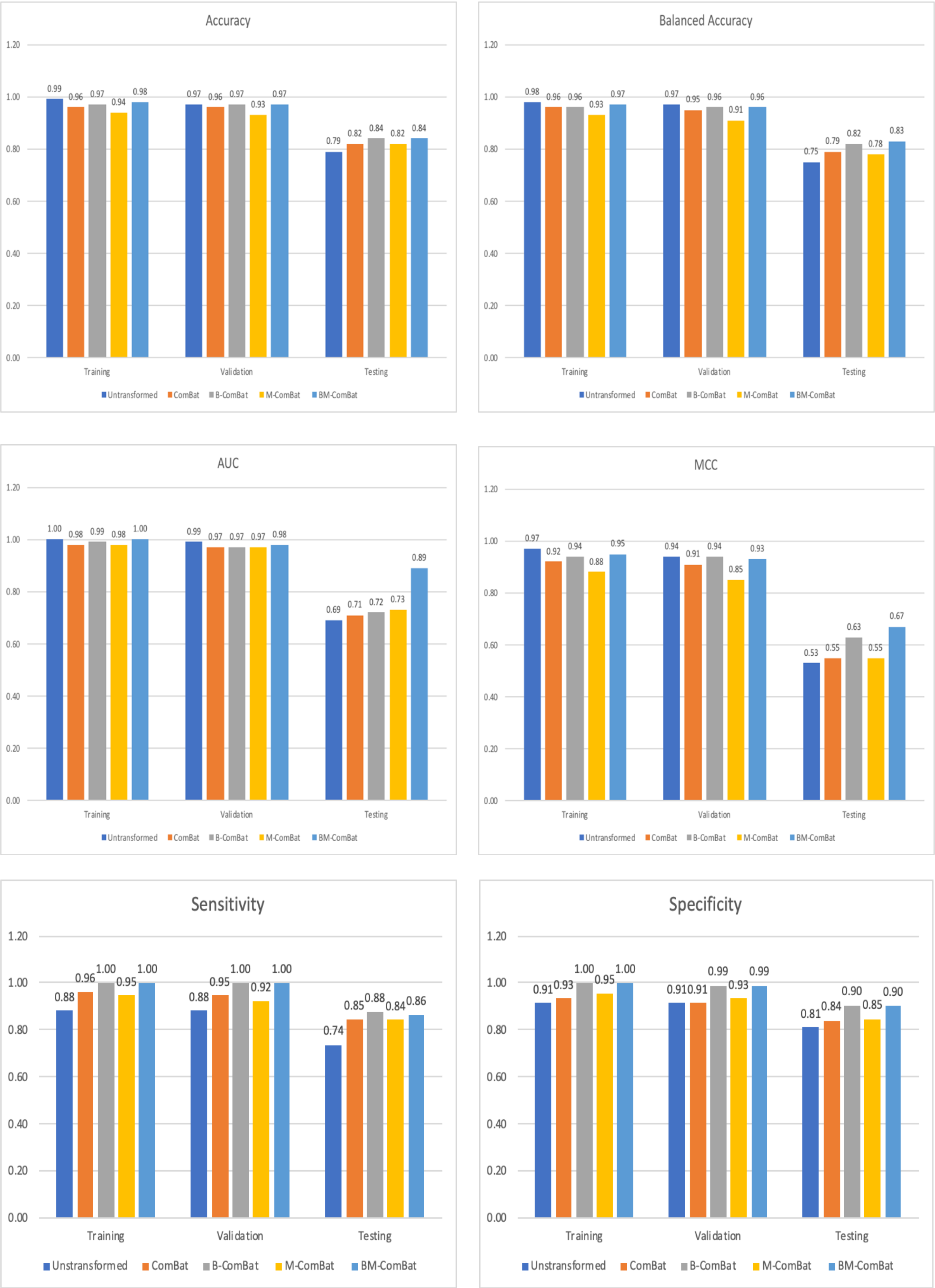

Supplemental Figure 10 - Performance metrics evaluation of predictive models in LALC data using SVM.

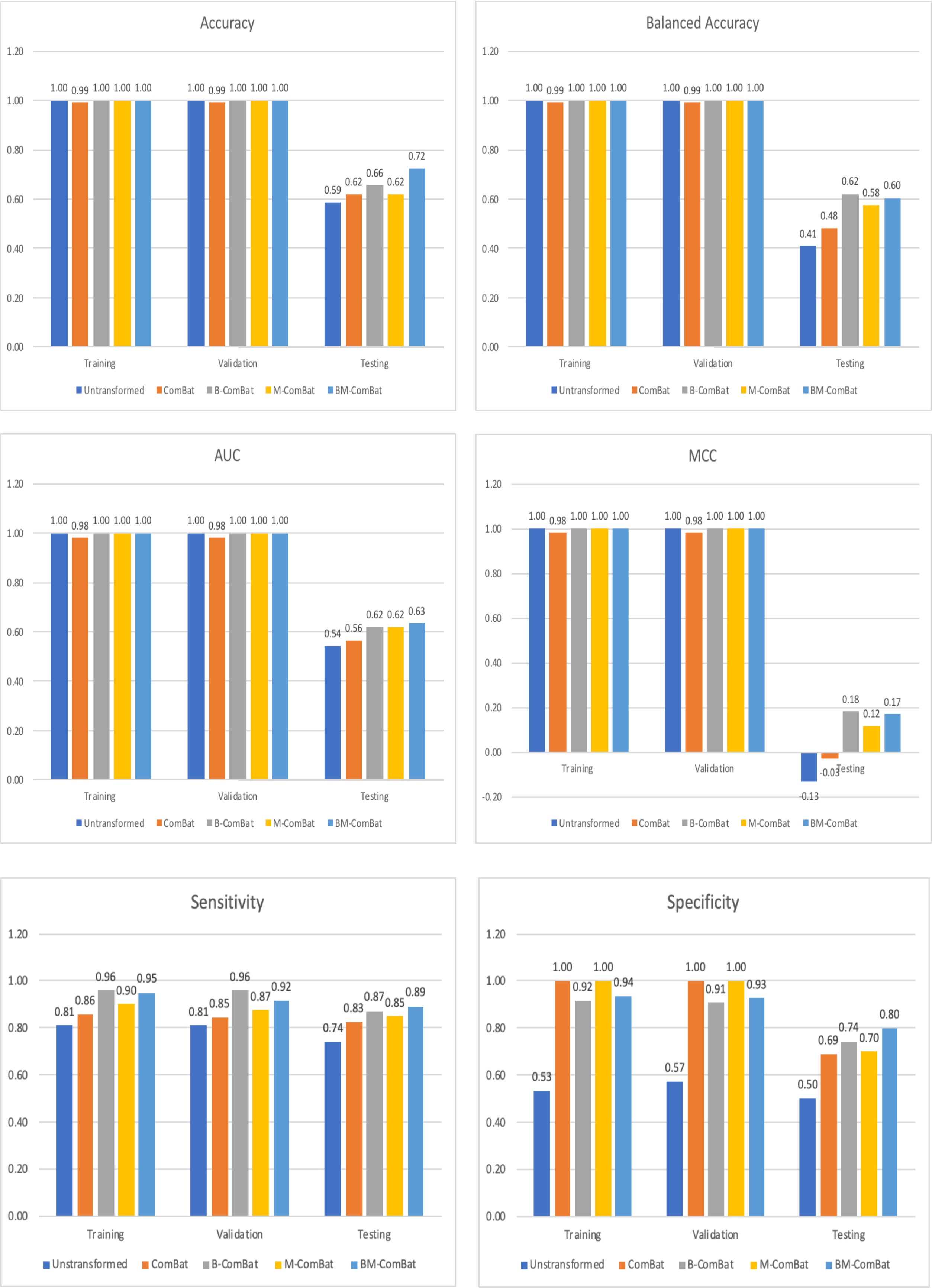

Supplement: Supplementary file 1 — Supplementary Information. [file 41598_2020_66110_MOESM1_ESM.pdf]
